# The patient perspective on diversity-sensitive care: a systematic review

**DOI:** 10.1186/s12939-024-02189-1

**Published:** 2024-06-05

**Authors:** Ewout Daniël Lieven Lauwers, Robin Vandecasteele, Michael McMahon, Stéphanie De Maesschalck, Sara Willems

**Affiliations:** 1 Erasmus University Medical Center, Dr. Molewaterplein 40, Rotterdam, South Holland 3015 GD The Netherlands; 2 Faculty of Medicine and Health Sciences, Department of Public Health and Primary Care, Research Group Equity in Health Care, Ghent University, University Hospital, Campus Entrance 42, C. Heymanslaan 10, Ghent, 9000 Belgium; 3 Faculty of Medicine and Health Sciences, Ghent University, C. Heymanslaan 10, Ghent, 9000 Belgium; 4 Faculty of Medicine and Health Sciences, Department of Public Health and Primary Care, Quality & Safety Ghent, Ghent University, University Hospital, Campus Entrance 42, C. Heymanslaan 10, Ghent, 9000 Belgium; 5 Centre for the Social Study of Migration and Refugees, Ghent University, H. Dunantlaan 2, Ghent, 9000 Belgium

## Abstract

**Background:**

The provision of diversity-sensitive care is a promising approach towards reducing health disparities. Recent criticism and a scientific gap demonstrate the need for the patient perspective on diversity-sensitive care. This systematic review aims to describe the patient perspective, including patient experiences, expectations, and satisfaction with diversity-sensitive care provided by healthcare providers.

**Methods:**

In December 2022 the Medline ALL, Embase, Web of Science Core Collection, Cochrane Central Register of Controlled Trials, CINAHL, PsycINFO and additionally Google Scholar were searched for original studies that described or measured patient expectations, experiences, and/or satisfaction, specifically focusing on cultural or diversity competence of healthcare providers. Analysis of the collected data was performed using a convergent mixed-methods design based on thematic synthesis.

**Results:**

From initially 5,387 articles, 117 were selected for full-text screening, and ultimately, 34 articles were included in this study. The concept of diversity-sensitive care was observed to comprise three components. The first component is focused on patient-centered care and includes competencies such as clear and direct communication, shared decision-making, individualized care, empathy, and consideration. The second component centers on providing culturally tailored information, adjusting care to cultural needs, working with interpreters, allyship, community partnerships, self-awareness, and cultural knowledge, and builds upon the first component. Across the first two components of diversity-sensitive care, patients have reported experiencing dissatisfaction and encountering shortcomings in their healthcare providers, sometimes resulting in the third and final component pertaining to provider care. This component underscores the importance of linguistic, ethnic, cultural, and gender concordance in delivering quality care.

**Conclusion:**

In conclusion, the patient perspective on diversity-sensitive care encompasses multiple components, from patient-centered care to concordant care. The components incorporate various competencies as communication skills, empathy, self-awareness and adjusting care to cultural needs. Patients reported experiencing dissatisfaction and shortcomings across all components of diversity-sensitive care provided by healthcare providers.

## Introduction

Health disparities are preventable differences in healthcare quality and/or outcomes between groups that reflect social inequalities [1]. Disparities are experienced by populations that have been disadvantaged by their social or economic status, geographic location or environment, such as ethnic minorities, people with limited language proficiency, sexual and gender minorities [1, 2]. Although discrimination, diversity, inclusion and equity are trending subjects of debate and policy nowadays, research on health disparities is not new at all. In fact, for over fifty years researchers have been trying to understand the impact of social inequalities, like social class [3] and ethnicity [4] on health, and how to overcome the health disparities they produce.

Despite years of research and knowledge gathering, the COVID-19 pandemic has demonstrated that social inequalities persistently contribute to disparities in health outcomes within European healthcare systems, in spite of several transnational anti-discrimination policies [5, 6]. Studies have indicated that certain population groups, particularly those having a migration background and individuals with low incomes, were more vulnerable to COVID-19 and have been disproportionately impacted by the disease, leading to higher mortality rates [7, 8]. As a result, the topic of health equity has not only gained significant attention in present time but has also become exceptionally pertinent. The pandemic has served as a reminder that, similar to the pursuit of health access, quality and efficiency, the pursuit of equity must be ongoing [7], necessitating a coordinated, multifaceted and interdisciplinary approach [8, 9].

In order to achieve more equitable health and healthcare, it is crucial to address and diminish disparities. They stem from a multitude of causes, are multifactorial and largely originate from factors external to the healthcare system itself [10]. In recent years, research focus has shifted from examining financial and insurance-related causes of disparities towards non-financial barriers that operate within the patient-doctor dynamic [10, 11]. This shift in focus of research has been driven by the recognition of the increasing diversity in society and the consequent need for cultural competence in healthcare [11]. Betancourt and colleagues [10] defined cultural competence as the ability to provide care to patients with diverse values, beliefs and behaviors, including tailoring the delivery of care to meet patients' social, cultural, and linguistic needs. In the conceptual framework of Campinha-Bacote and colleagues [12], cultural competence encompasses cultural awareness, cultural knowledge and cultural skills. Cultural competence has since long been recommended as a strategy to address health disparities, and there is ample evidence supporting the effectiveness of this approach [13–16].

In recent years, the conceptual framework of cultural competence has faced criticism. It has been argued that cultural competence, as a static concept that can be achieved solely through training in knowledge, skills and attitudes, neglects the systemic mechanisms and dynamics present in medical encounters [17]. There are concerns that cultural competence can be ethnocentric in its orientation, perpetuating long-standing biases and failing to acknowledge the pervasive nature of supra-individual contributing factors such as systemic racism [18]. Researchers are now questioning the framework’s true ability in addressing health disparities [18, 19]. Furthermore, despite twenty years of emphasis on cultural competence, health inequities continue to exist, as the COVID-19 pandemic showed [20]. This failure is attributed to the neglect of systemic mechanisms within the cultural competence approach, which the pandemic has brought to light [21]. In response to these criticism, cultural safety and cultural humility have emerged as alternative, more dynamic approaches. These involve ongoing and reflective processes, with a focus on ‘critical consciousness’ and building honest and trustworthy relationships [22, 23].

For a considerable period, literature has predominantly focused on ethnicity and culture as determinants of diversity, and thus of focus in cultural competence [24]. However, based on current insights on superdiversity and intersectionality, there is need for broader interpretation. Superdiversity refers to the increasing diversity in society in general but also within cultural groups. It recognizes that even within a single cultural group, individuals have vastly different backgrounds, experiences and needs, both culturally and socially [25]. The theory of intersectionality places emphasis on the interaction of different social identities and experiences of (institutional) discrimination [26]. Cultural competence training often emphasized understanding and accommodating cultural practices and beliefs of specific ethnic groups [10]. However, with the recognition of superdiversity, this approach has become limited. There is need for understanding of culture beyond ethnicity. Although ethnicity remains a significant aspect of culture for many people, increased diversification of Western society complicates categorization of culture, especially solely based on ethnicity [25]. For example, in large, superdiverse cities, people from different ethnic backgrounds may share more similarities in beliefs and values than they do with people from the same ethnic group living in rural areas. Furthermore, the interaction of various social sub-identities is essential to consider, more than only the culture-ethnicity interaction. Gender identity, socioeconomic status, education level, geographical location and other social determinants also play a role in shaping cultural experiences [26]. Therefore, when addressing cultural competence, it is important to consider the intersection of various identities that create diversity.

Researchers and clinicians have devoted considerable efforts to developing, debating and defining these concepts over the years [19]. Historically in literature, cultural competence has been dominant. With the previous criticism regarding a broader interpretation of cultural competence in mind, alongside with aforementioned criticism regarding cultural competence being static and neglecting systemic mechanisms, the choice is made to employ the term 'diversity-sensitivity' to advocate for an awareness that extends beyond mere considerations of culture and ethnicity.

The criticism directed towards cultural competence highlights the need for an interpersonal stance that is other-oriented [19]. In cultural competence, patient-centeredness is very important [27]. However, the perspective of the patient is almost not heard in this debate. There is a noticeable research gap concerning the patient perspective on diversity-sensitive care [28–30]. Earlier literature reviews have emphasized the urgent need to address this gap, as there is a limited number of articles focusing on the patients’ viewpoints [31, 32]. The absence of the patient perspective is concerning, particularly given the current shift towards culturally safe care and the significance of incorporating patient views in defining cultural safety [32]. Therefore, a systematic review was conducted to describe the dimensions of the patient perspective on diversity-sensitive care provided by a healthcare provider, through examination of patient expectations, experiences and satisfaction, with a focus on the interpersonal relationship.

## Methods

This systematic review was conducted in accordance with the Cochrane Handbook of Systematic Reviews and the Preferred Reporting Items for Systematic Reviews and Meta-Analysis (PRISMA) guidelines. The protocol for the study was registered with the International Prospective Register of Systematic Reviews (PROSPERO) under registration number CRD42023385433.

### Search strategy

The search strategy for this systematic review was developed in collaboration with the Knowledge Centre for Health Ghent (KCGG) of the University of Ghent and the Medical Library of Erasmus University Rotterdam, under the supervision of a biomedical information specialist. On December 1st, 2022, a comprehensive search was conducted using MEDLINE ALL, Embase, Web of Science Core Collection, Cochrane Central Register of Controlled Trials, CINAHL, PsycINFO and Google Scholar. Both free text and keywords were used in title and abstract searches, and adjacency operators were used due to the heterogeneity in terminology. The search strategy was adjusted for each database, with a focus on two main concepts: *diversity sensitivity* and *patient perspective*, as elaborated in Table 1. Studies with a primary focus on education were excluded using the Boolean operator ‘NOT’.

**Table 1:** Search strategy

**Diversity sensitivity**		**Patient perspective**	
Cultural competency		Patient	Satisfaction
Cultural sensitivity		Client	Perspective
Transcultural	Healthcare	Minority	Expectation
Cross-cultural	Service	Refugee	Experience
Multicultural	Humility	Migrant	Perception
…	…	…	…

The full syntaxes of the search strategy, tailored for each database, are available in Appendix 1.

### Eligibility criteria

The inclusion criteria for this systematic review were as follows:

1. Studies investigated the patient perspective, including descriptions or measurements of patient expectations, experiences, barriers, and/or satisfaction.
2. Studies utilized a qualitative study design, such as in-depth interviews or focus groups, or a quantitative or mixed-methods study design using surveys.
3. Studies focused on the cultural or diversity competence of healthcare providers, specifically exploring the delivery of culturally or diversity-competent or diversity-sensitive care.

The following exclusion criteria were employed:

1. Studies that lacked the patient perspective, a focus on diversity-sensitive care or elaboration or interpretation of factors contributing to ratings of cultural competence.
2. Studies that did not pertain to Western healthcare (member countries of the Organization for Economic Co-operation and Development).
3. Studies that were not original research articles, such as reviews, dissertations, expert opinions, letters, case reports, or background articles.
4. Studies that were published before 1990.
5. Studies that did not have full-text available.
6. Studies with an exclusive focus on social determinants other than ethnicity.

### Screening process

The results of the search strategy were imported into Endnote, and duplicates were removed. The title and abstract screening was completed by the first author. To ensure consistency in application of eligibility critiria, the involved team members had discussions to address and clarify potential differences in interpretation. Afterwards, two team members independently assessed all remaining full-text articles with the aforementioned eligibility criteria in mind. The conflicts in full-text screening were resolved throughout deliberation until consensus was reached. When necessary, a third team member was consulted to make a final decision.

### Data extraction

Data was collected from the original articles using a predetermined table, designed by the first author. The extraction was completed by two team members independently, with deliberation until consensus was reached. The data table consisted of the following categories: first author, year of publication, country of origin, study design, analysis, cultural competence scale, survey used, sample size, population of interest, additional patient characteristics, provider characteristics, care setting, outcome category and outcomes.

### Quality assessment

Methodological quality was evaluated by two team members independently after completion of the screening and data extraction process. In cases where discrepancies arose, consensus was reached through discussion. The Critical Appraisal Skills Programme (CASP) was utilized for qualitative research studies, and participants could respond with one of three possible answers: 'Yes', 'Unclear', or 'No'. Similarly, the Joanna Briggs Institute (JBI) Critical Appraisal Tool was used for quantitative studies, with response options of 'Yes', 'Unclear', 'No', or 'Not Applicable'. For mixed-methods research, the Quality Assessment Tool for Studies with Diverse Designs (QATSDD) was employed, with responses ranging from 0 to 3. Following completion of the evaluations, the results were standardized to allow for comparison across study designs, resulting in a scale from 0 to 10. In more detail for the CASP and JBI tools, responses of 'Yes' were assigned a score of 1, 'Unclear' a score of 0.5, 'No' a score of 0, and 'Not Applicable' was designated as a dropout. To facilitate comparisons across all study designs, the QATSDD scoring was reduced from an 0 to 3 to a 0 to 1 scale.

### Analysis

In order to integrate qualitative, quantitative and mixed-methods data, a convergent mixed-methods design was followed [33]. First, the qualitative data was analyzed. Synthesis consisted of an inductive thematic synthesis, as developed by Thomas and Harden [34]. The synthesis method is threefold. First, concepts in original research were identified by the principal researcher. Second, combination of constructs led to the formation of descriptive themes, still close to primary data. Finally, by further analyzing and interpreting the themes, new constructs were developed. Throughout the thematic synthesis, there was consultation with the research team for the identification, analysis and interpretation of concepts, constructs and themes. In consultation sessions, ideas were visited and revisited until consensus was reached. Second to the qualitative synthesis, a narrative synthesis of quantitative and mixed-methods data was performed. After contextual consideration, the data was incorporated within the fitting themes that the qualitative synthesis yielded.

## Results

### Search results

**Fig. 1:**
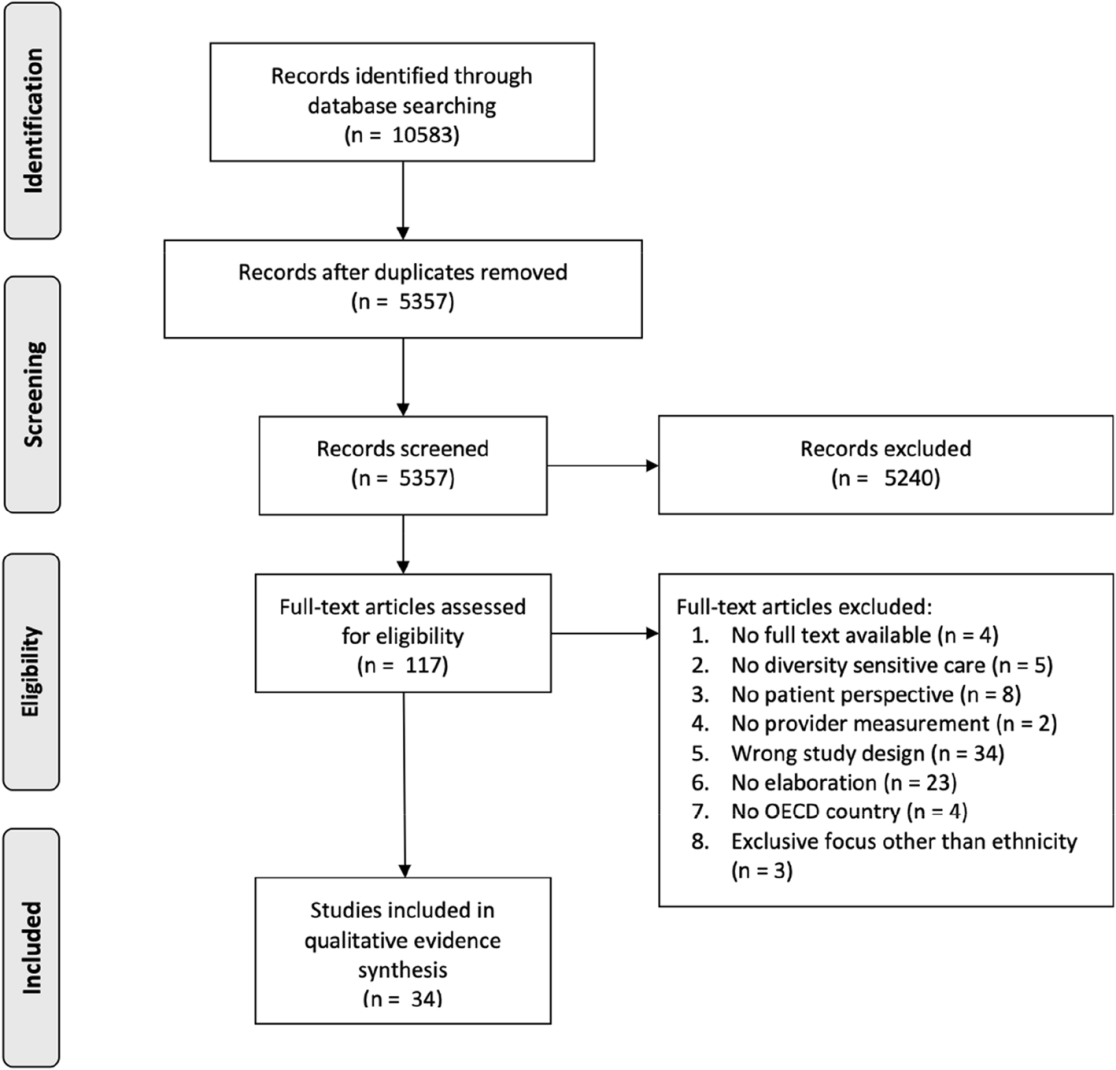
Study selection

The search through database exploration produced a total of 10,583 results. Subsequently, 5,357 single records were identified after removal of duplicates and screened on title and abstract. Out of these, 5,240 records were excluded based on initial screening results. During eligibility assessment, the main reasons for exclusion were inappropriate study design (34 studies) and insufficient elaboration on factors contributing to the measurement of cultural competence (23 studies). Ultimately, 34 studies were deemed eligible for further analysis. The detailed screening process is illustrated in Fig. 1. More details on the database searching can be found in Appendix 1.

### Study characteristics

Of the included studies, 23 are categorized as qualitative, 9 are cross-sectional, and two studies feature a mixed-methods research design. The majority of the research was carried out in the United States (18), Europe (6), Canada (5), and Australia (4) and New Zealand.

In the context of cross-sectional research, the Community Assessment Instrument (CAI), Client Cultural Competence Inventory (CCCI), Cultural Competency and Services Provision Questionnaire (CCSPQ), Cultural Competence Assessment (CCA), and Physicians Cultural Competence for Patient Satisfaction (PCCPS; administered twice) were employed as tools to evaluate the level of diversity-sensitivity among healthcare providers.

The majority of the studies selected the patients based on their ethnic background (ethnic minority individuals or a heterogeneous group of ethnic minority and majority individuals). Few studies used the socioeconomic or religious background, sexual orientation, or mental health status as criterium for inclusion in the study. Furthermore, four studies did not define a specific patient population. The number of patients included varied between 4 and 22,864 individuals. The median number of participants across all included studies was 32.

The majority (24 studies) of studies under review reported on the patients’ perspective on physicians’ cultural competence. In a limited number of studies also nurses, therapists, and other allied healthcare professionals were explored. Furthermore, the care setting varied considerably, encompassing predominantly inpatient hospital care (9 studies), as well as home-based care (5 studies), prehospital acute care (2 studies), emergency care (2 studies), primary and community-based healthcare (7 studies), mental health care (3 studies), and unspecified care settings (6 studies).

The outcomes studied in the included studies were predominantly *patient expectations* and *patient experiences*, in addition to ratings of *cultural competence*, *clinical outcomes*, and *satisfaction*.

Assessment of the methodological quality of included studies showed on average mediocre quality, with 10 studies scoring a 6 out of 10 or lower, and 13 studies scoring a 8 out of 10 or higher. Additional details on the quality assessment of all included papers can be found in Appendix 2. Specific characteristics of the included studies are delineated in Table 2.

**Table 2:**
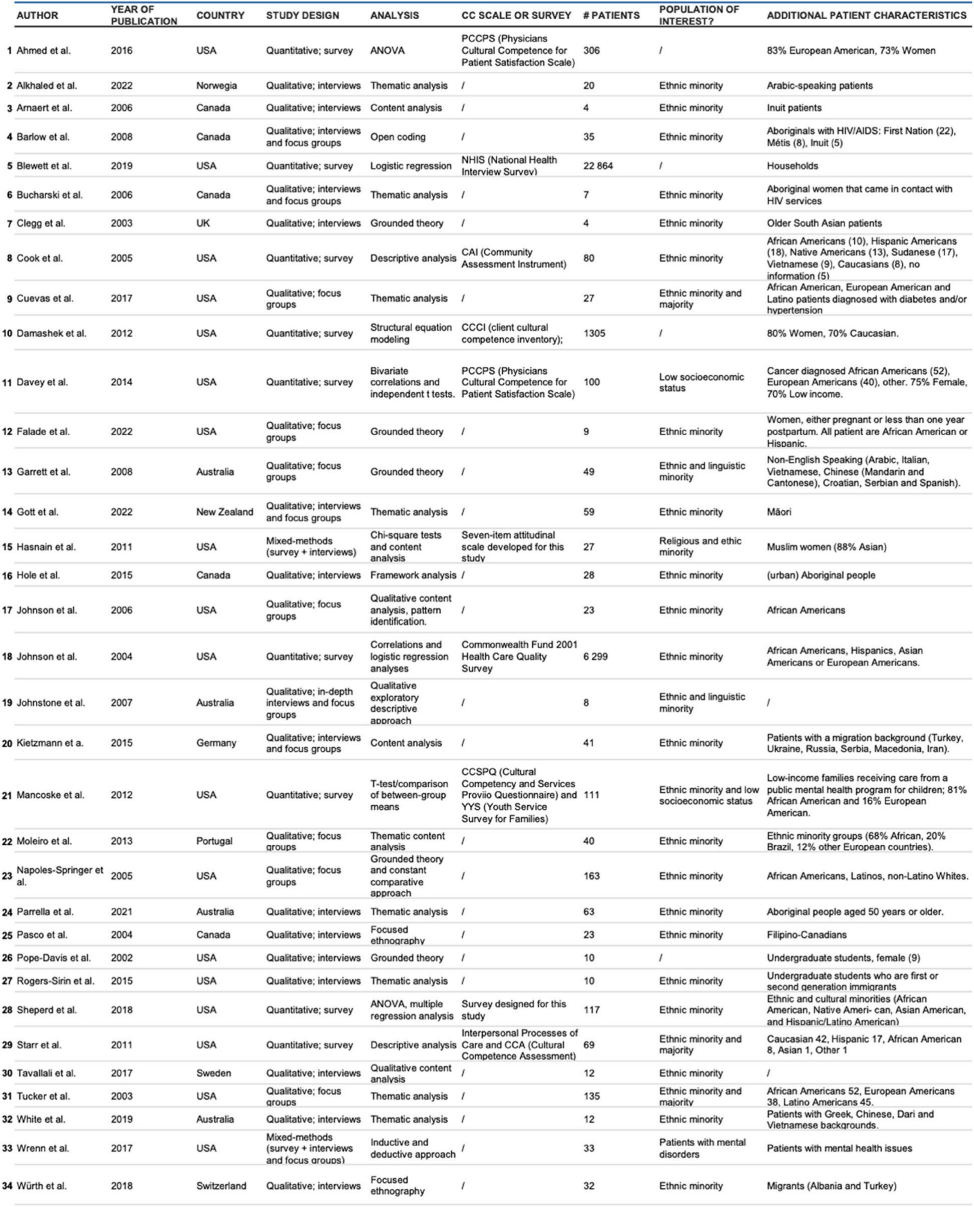
Study characteristics

### From patient-centered care towards diversity-sensitive care

**Fig. 2:**
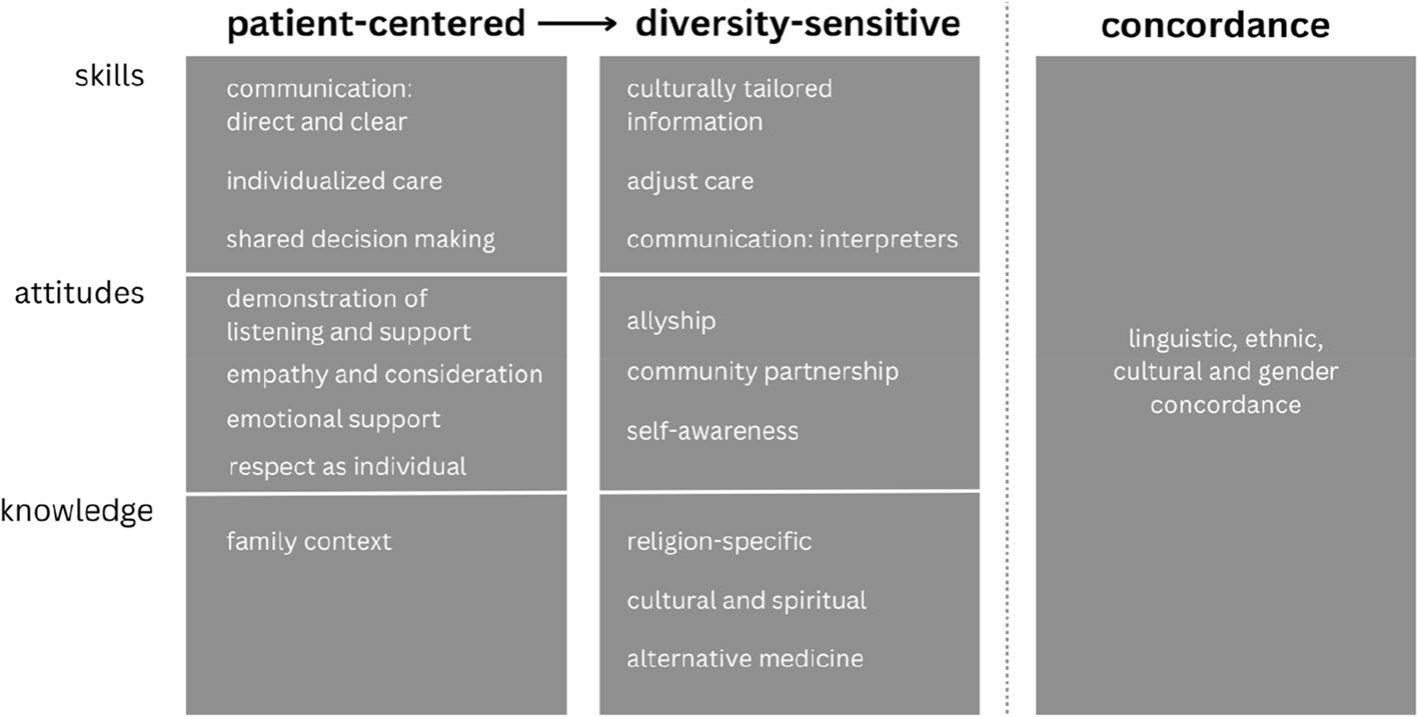
The patient perspective: from patient-centered care towards diversity-sensitive care

The primary outcomes are depicted in Table 3. The thematic analysis of the included studies identified that diversity-sensitive care, from the patient’s perspective, can be conceptualized into three distinct components (see Fig. 2). The initial two, patient-centered care and diversity-sensitive care, are interconnected and encompass knowledge, attitudes, and skills. Diversity-sensitive care builds on patient-centered care, and requires more complex and targeted competencies that are specifically relevant to diversity-sensitive care. Patients’ experiences in deficiencies in these two components lead to a preference for concordant care, where patients desire a healthcare provider who shares their identity such as gender or ethnicity [35], as depicted in the third component.

**Table 3:**
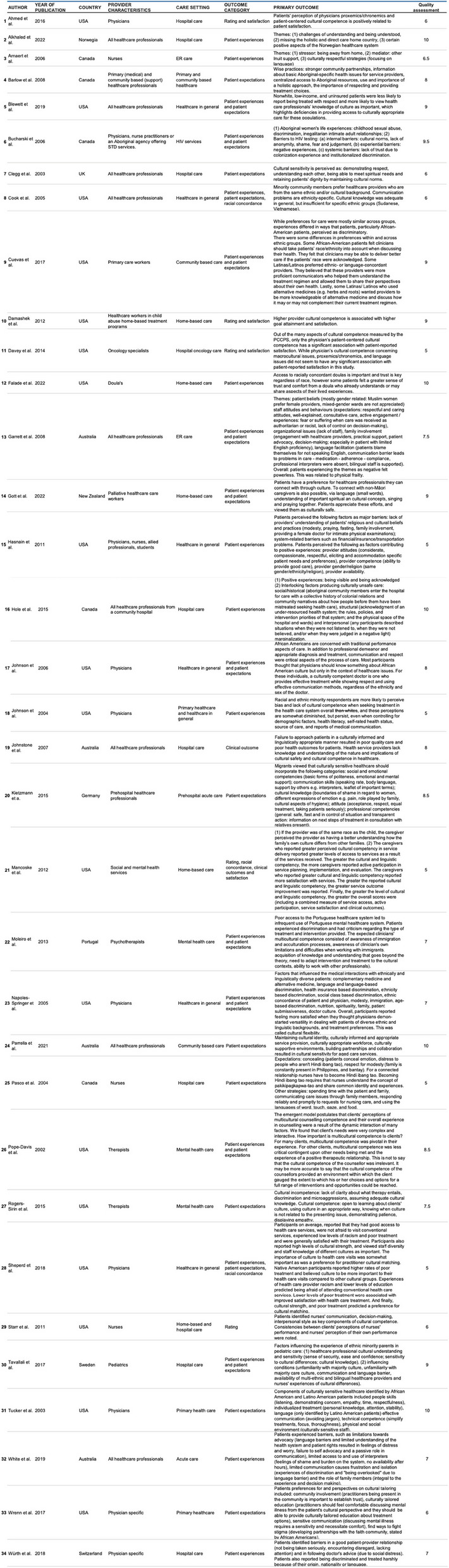
Primary outcomes

The three components are presented in a sequential way, accompanied by an exploration of the mentioned competencies (skill, attitudes and knowledge) along with reported experiences.

### Patient-centered care

#### Competence: skills, attitudes and knowledge

##### Attitudes

Patients mention three distinct yet interconnected attitudes that healthcare providers could adopt to deliver better patient-centered and diversity-sensitive care. Firstly, patients express their desire for healthcare providers who are friendly, approachable and accessible. Secondly, patients emphasize the importance of being heard and valued, urging healthcare providers to show more consideration, responsiveness and take their concerns seriously. Lastly, patients report a desire for healthcare providers who are considerate, compassionate, and demonstrate genuine empathy [36–38]. These three attitudes converge to fulfill what some patients describe as a need for emotional support [37, 38].

Additionally, many patients highly value healthcare providers who show respect towards them as individuals. Respect is defined as the recognition of personal rights to hold beliefs, make choices, and act in accordance with personal values [39]. Many patients perceived respect as a crucial aspect of care [30, 37, 40–44].

“It is important people understand my religion and are respecting me for who I am. I am a well-educated person, high standing in Pakistan. I taught children to read and write Urdu but that does not count for anything here” [42].

##### Skills

The primary skill highlighted by patients is shared decision-making. Charles and colleagues [45] define shared decision-making as a collaborative process in which both physician and patient being share information and strive to reach consensus on the preferred treatment. Most patients reported placing high value on shared decision-making [44, 46, 47]. It is recommended that physicians strike a balance between providing advice and making proposals while respecting patients’ autonomy through exploration and negotiation [43]. However, not all patients report being equally involved in the decision-making as they desire. For example, one study pointed out how Hispanics and Asians were less likely to report being as involved as they wished, compared to Whites and African-Americans [48].

Another skill valued by patients, somewhat overlapping with the above, is the healthcare providers’ ability to individualize care and treatment. Patients express greater satisfaction when providers demonstrate attentiveness by remembering their names and specific details of their case, showing sensitivity towards their financial concerns, and seeking their perspectives and viewpoints [36, 38, 49].

“The doctor (female) was listening to me…. she took my personal hesitations and preferences into account. She explained insurance coverage details.” [36].

The third skill in the context of patient-centered care relates to communication. Patients value healthcare providers who explain information sincerely, directly and without the use of jargon [38]. Clear and effective communication are perceived as very important [47, 50].

The final skill identified by patients relates to providing comfort during medical consultations. Patients have consistently reported that healthcare providers who exhibit patience during consultations contribute to a sense of comfort [29]. Patients expect healthcare providers to possess the skill of instilling confidence, ease and comfort during their medical encounter [50]. Furthermore, the effective and comfortable use of physical space and time were also correlated as contributing factors to patient satisfaction [49]. However, another study found the correlation between physical space and time and patient satisfaction to be insignificant [51]. Nevertheless, in both studies, patient-centered competence was identified as the most critical factor contributing to patient satisfaction [49, 51].

##### Knowledge

Several studies have examined patients' expectations regarding healthcare providers' knowledge of their perspectives on family involvement and the family context [36, 37, 44, 52, 53]. Some patients have indicated that they consider their family members to be integral to their healthcare experiences [52]. This viewpoint may stem from the involvement of family members in facilitating communication, such as serving as interpreters for patients [52]:

“If I don’t understand, I ask to stop as my daughter is coming” [52].

Patients have articulated their desire for the close involvement and availability of family members, even in the decision-making process [37, 52]. They further highlighted the importance of familial solidarity and expected healthcare providers to be well-informed about this aspect [37]. However, patients also acknowledge that such involvement may potentially lead to prolonged hospital stays and delays in procedures [52].

### Experiences

Many patients described situations in which healthcare providers did not listen nor believed them [54, 55]. For some patients, the failure to address patients’ concerns in accordance with their expectations resulted in failure to be regarded with due seriousness [55]. Other patients reported feelings of ignorance or neglect [29, 52, 54]. Consequently, certain patients found themselves lacking information pertaining to their diagnosis and treatment [29, 54], leading to sensations of distress, confusion, and discomfort [29].

“They don’t pay enough attention. They assume that I am an idiot – not worth it.” [52].

Ethnic minorities, individuals with low income and uninsured patients have consequently reported receiving less respect from healthcare providers [56]. Furthermore, a significant number of patients have shared their experiences of discrimination within healthcare settings [29, 55, 57–60]. Patients attribute such perceived discrimination to factors like ethnicity or language [55].

### Diversity-sensitive care

#### Competence: attitudes, skills and knowledge

##### Attitudes

Healthcare providers who are not culturally concordant with their patients can provide culturally safe spaces. The foundation for this approach lies in the continuous effort to establish a connection with the patient. By embracing the following attitudes, patients believe that healthcare providers can transition from being approachable and showing empathy and emotional support to fostering culturally safe spaces. To achieve this, healthcare professionals should display comprehension by integrating their knowledge of cultural concepts, customs, and rituals. Additionally, engaging in activities such as singing and praying together, particularly in situations like end-of-life care, can foster a sense of empathy and cultural safety [46]. However, not all efforts need to be arduous or time-consuming. Simple gestures like using small words from patients’ native language are highly appreciated [46]:

“ … they greeted us with a “Kia ora”, (hello!)… ah… “Ka kite”, (see you/goodbye) … [the] Pākehā [nurses]. And they were using, you know, Māori kupu (words)… which is quite funny like [laughs]… you know. But they’d always come out with… you know, “We’re here for not only you, but the family, and the whānau”, and use those sorta words, to… connect with us… Yeah. They did very well…” [46].

Furthermore, visibly demonstrating inclusivity or using culturally sensitive materials, can offer reassurance of cultural safety and competence [38]. Unfortunately, failure to display inclusive materials can result in feelings of exclusion:

“Every time you walk into one of these facilities you see White doctors’ pictures up and you see all these paintings up. But you’ll never see the first man that did heart surgery which was a Black man. You don’t see that up here” [38].

These attitudes can be understood within the framework of allyship [46], which involves combatting injustice and promoting equity through active advocacy [61]. The National Institute of Health describes allyship as, “When a person of privilege works in solidarity and partnership with a marginalized group of people to help take down the systems that challenge that group’s basic rights, equal access, and ability to thrive in our society” [62]. Such attitudes play a pivotal role in patients experiencing their healthcare providers as (cultural) allies with proactive behavior regarding safety [46].

Next to allyship with the individual patient, building partnerships with patient’s communities is highly valued by patients. Patients meeting their physicians at, for instance, community events or in church are possibly more comfortable discussing difficult health topics when in the consultation room. This could be attributed to a reduction in stigma surrounding seeking treatment, which, in turn may encourage open communication [63]. Stronger partnership between healthcare providers and cultural networks and organizations could provide reassurance for patients that traditional services in medical care are available for those who request them [41]. The involvement of healthcare providers in the community of the patient is a strong predictor of self-reported patient satisfaction [64].

“We want to see our physicians in our neighborhood” [63].

Awareness and sensitivity are considered key aspects in providing diversity-sensitive care [50, 59]. It is shown that healthcare providers are regarded as culturally sensitive when they acknowledge and respect cultural differences, demonstrate awareness of the impact of these differences, and act accordingly [50]. Showing respect for cultural differences also predicts self-reported patient satisfaction [64]. Specific aspects of cultural differences that need the attention of healthcare providers, are cultural specific issues and hardships that patients experience. Healthcare providers should be mindful of the disadvantages (e.g. financial, housing) that patients can experience because of these differences [65]. In addition to being aware of patients' backgrounds, self-awareness is considered fundamental. According to Lu and colleagues [66] (cultural) self-awareness refers to an individual's understanding of how their culture has shaped their identity. Those with high cultural self-awareness recognize how their cultural experiences have influenced their values and behaviors [66]. Patients have suggested that providers should be aware of their own limitations when working with non-concordant patients, acknowledge their own biases, stereotypes and negative emotions [59].

By reasoning and acting from their own perspective, healthcare providers sometimes judge behavior or beliefs that patients regard as part of their culture, as pathological or problematic. Patients report that this judgement creates unsafety [29].

##### Skills

In intercultural clinical encounters, healthcare providers should be able to distance themselves from their own perspective, and be skilled in discussing illness from the patient’s cultural perspective. Furthermore, they should adapt information to the patient’s background, e.g. about advice and treatment options, providing so called culturally tailored education [63].

With appropriate cultural knowledge of and awareness regarding possible important needs and values of patients, providers should be able to adjust care in order to accommodate these values and needs. For example, dietary restrictions, special needs during fasting and the need for same-gender providers require special attention, consideration and accommodation by healthcare providers [36]. Healthcare providers should differentiate in care between people depending on their background as patients expressed their need to stay connected to their culture [30].

Moreover, patients sometimes consider communication as essential in providing holistic and culturally safe care [46], since it has been noticed that problems of communication intensify the perception of cultural misunderstanding [28]. It is suggested that in cross-cultural care, translating and connecting between the perspectives of patient and healthcare provider are viewed as effective communication [46]. Some patients feel that in communicating effectively, healthcare providers demonstrate their knowledge and sensitivity to the patient’s health beliefs and practices [43]. Numerous patients contemplated good communication fundamental for self-advocacy, understanding and active participation in care [52]. However, communicating across a language barrier poses an enormous challenge [42]. To overcome this difficulty, there are multiple strategies that healthcare providers could adopt. Patients reported that interpreters are crucial for achieving effective communication across a language barrier [37, 44, 52].

“If there can be interpreters present – that will be the best thing” [52].

Unfortunately, patients additionally reported that access to and presence of professional interpreters varied widely [52]. For instance, with inpatient and emergency care, patients regarded the absence of interpreters as only logical due to the acute setting of some medical encounters and unscheduled consultations [44]. Furthermore, patients believed interpreters are not available after hours [52]. Other strategies for interpretation were also mentioned by patients, bringing a family member, bilingual staff [37] or English as bridge [28]. Nonetheless, some patients did mention they would rather not have their relatives act as interpreters [28]. In case of low language barriers, patients reported to benefit from healthcare providers adjusting their speaking rate and using body language [37].

Not only do language barriers present challenges to effective communication, it has also been suggested that they can impose significant emotional distress on patients. Failure to communicate in a mutually understandable language frequently gives rise to feelings of worry, uncertainty and frustration. In addition, patients reportedly feel more isolated and discriminated in comparison to patients who speak a mutual language [52].

“Nurses would go more often to the patients who spoke English” [52].

Moreover, some patients reported feelings of shame and burden to the healthcare system and themselves because of language barriers. More so, they felt that their limited language skills could be a hindrance to their own cross-cultural care experience [50, 52]. Patients sometimes feel that they simply have to endure the language barrier [52].

“Maybe that’s my problem, because I can’t speak Swedish well, so I can’t have a good relationship with them. They are very kind, but I feel ashamed to talk to them” [50].

Two cross-sectional studies described the correlation between the competence on language issues as part of cultural competence and patient satisfaction as not significant. In both studies, patient populations consisted of a homogeneous English-speaking population [49, 51].

##### Knowledge

Patients emphasize the significance of healthcare providers having knowledge about their culture [43, 54] as it is crucial for them to feel understood [33, 59]. Specifically, religious needs such as modesty, fasting, praying, and dietary restrictions are considered important for healthcare providers to be aware of [36]. Religion holds a fundamental place in the lives of some individuals and can have an impact on their health and hospitalization experiences. In the study of Johnson et al., religious patients often expect healthcare providers to acknowledge, understand, and accommodate their religious beliefs [42].

“A few years ago, I had to consult a gastroenterologist. My doctor was male, and he did my rectal examination in his office…. there was no female nurse nearby. It left a negative impact on me. Being a woman and above this a Muslim, I felt it was very negative to be treated by a male doctor in the absence of a female” [36].

In addition to religious knowledge, patients could feel that healthcare providers should understand their ideological and spiritual needs. Patients sometimes ask for inclusion of traditional teachings and practices in the clinical encounter [65] and understanding of important spiritual concepts [46] e.g. waka (canoe; Aboriginal) to help explain care in the event of death. Patients could feel that understanding of their traditions and beliefs is essential to them, and that acknowledgment of differences by healthcare providers is the first step [42].

Apart from specific cultural customs, rituals, concepts and practices, healthcare providers should know that the value placed upon “universal” needs such as e.g. privacy and personal space can vary between cultures [42]. For some patients, alternative or traditional medicine can be part of their culture and belief system. Patients might want healthcare providers to be open to this and integrate traditional medicine, such as massage therapy, chiropractic, herbal treatment and acupuncture into their treatment plan [54, 58]. Furthermore, adequate knowledge about alternative medicine in order to be able to have a serious conversation about it, e.g. to see if alternative treatment does not interfere with the Western medical treatment, is something that patients may wish for [57]. However, other patients feel that because their healthcare providers are not knowledgeable, it is more comfortable not to discuss it [43].

“Some felt that physicians stopped listening when they brought up alternative medicine, which they felt was disrespectful to their beliefs” [58].

Although understanding of culture is deemed important by some patients, there is wide diversity between cultural groups and within groups. Patients can identify themselves with a culture, but this does not imply that they follow traditional ways or are knowledgeable of traditional customs. Patients emphasize they do not want to be stereotyped because of their identification with a group [41]. However, not all patients expect healthcare providers to have detailed knowledge of their culture. Some patients realize that it is impossible to know all traditions, religions or cultures, given the contemporary superdiversity [50]. Patients reported that in their experience providers did not have sufficient understanding of religious and cultural beliefs and practices [36].

### Linguistic, ethnic, cultural and gender concordance

A recurring theme across the included studies is patient and healthcare provider concordance. Minority populations have expressed a preference for healthcare providers who share their identities, such as ethnicity [67]. Without a shared identity, patients feel that they would conceal emotion and undergo more distress [68]. Patients have suggested that shared experiences may contribute to the sense of comfort experienced during medical encounters [65]. More specifically, patients have provided insight into their preference for receiving care from providers who share their ethnic, linguistic, cultural, or gender background and have articulated the reasons behind such preferences. A preference for language concordance arose from communication being more effective in concordant clinical encounters [57] and experiencing greater satisfaction [58].

“I think the, the majority of us try to look for Latino doctors, because first of all the communication that I think more than anything that’s really indispensable. Just understanding each other. And by understanding each other, the results of the doctor will be better, because they know exactly the problem we have. At least I, where I go, they’re all Hispanic. And I feel good, I feel good going there” [57].

Some patients surpassed the barrier of a shared language and expressed a preference for shared culture [46, 65, 67, 69] or ethnicity [53, 57, 58, 67, 70]. Patients believed that a shared culture or ethnicity is preferable because it enhances healthcare providers’ understanding and knowledge of patients’ backgrounds [57]. Furthermore, certain patients expressed a preference for gender-concordant care, as gender plays a significant role in cultural understanding [54, 69]. Some patients specifically found gender concordance important when discussing intimacy or undergoing intimate physical examinations [36, 37]. Moreover, patients expressed negative sentiments towards mixed-gender hospital wards [44].

Yet, the desire for concordance between healthcare provider and patient was not universally shared. Some patients believe that the same level of understanding can be attained even without concordance, through principles such as equality and mutual respect [69].

“I think a very important thing is to have a very equal relationship, where there’s mutual respect. I think those things transcend things like race and gender and age because if you’re able to establish a relationship like that with someone, [they] could be anybody really” [69].

## Discussion

### Summary of the results

In summary, the patient perspective on diversity-sensitive care can be summarized in three components. Firstly, patients expect healthcare providers to possess patient-centered competencies, which they consider a fundamental requirement for diversity-sensitive care. These competencies include skills such as shared decision-making, personalized care, and effective and transparent communication. Additionally, patients value attitudes that demonstrate concern, active listening, empathy, emotional support, and respect. Furthermore, providers should be knowledgeable about the patient’s family context. In the second component, that builds upon the first component, patients highlighted specific attributes that are particularly relevant to diversity-sensitive care. These skills involve providing culturally tailored information, adapting care to cultural needs, and effectively addressing communication challenges, including language barriers through appropriate collaboration with formal and informal interpreters. Attitudes that patients appreciate in healthcare providers include commitments to allyship and community collaboration, as well as self-awareness. Finally, patients expect healthcare providers to be knowledgeable about certain religious, cultural and spiritual needs, customs, practices and values. This understanding is essential for providing diversity-sensitive care. The third component emerges from patients experiencing shortcomings in the first two components. For some patients, achieving the desired level of understanding and comfort in healthcare settings was only possible through concordant care. Patients expressed the need for language, ethnic, cultural, and gender concordance in their healthcare interactions.

Comparison of the patient and healthcare providers’ perspectives on diversity-sensitive care reveals both logical similarities and interesting differences. Both patients and healthcare providers mention competencies according to Camphina-Bacote’s model, with categorization into attitudes, skills and knowledge [71, 72]. However, a considerable difference is that patients value cultural safety, as it is reflected in reported attitudes as allyship, sensitivity and self-awareness, whereas healthcare providers scarcely mention cultural safety at all [72]. The notions of allyship and self-awareness, however, have previously been founded to contribute to mitigating health disparties [73]. The self-reflexivity and dynamic aspect of some of the competencies mentioned also fit the concept of cultural humility better than cultural competence [74]. Apparently, patients do not differentiate between cultural competence, cultural humility and cultural safety, but simply expect an integrated approach from healthcare providers towards diversity-sensitive care. However, many articles do differentiate between cultural competence and cultural humility, as cultural humility is often viewed as an alternative approach to cultural competence [19]. In our view, the patient’s perspective does not support this. Instead, it supports the synergetic relationship mentioned by Campinha-Bacote [19]. Whether cultural competence needs redefinition to integrate cultural humility and cultural safety or they must be viewed in apposition remains unclear [75]. What is clear, nevertheless, is that the current definition of cultural competence, and application of only cultural competence, does not satisfy patients’ needs completely.

The extent of the relationship between patient-centered care and culturally competent care in research is subject to significant variation. Some see cultural competence as an essential skill set to acquire in order to provide effective patient-centered care to all patients [27]. Others see culturally competent care as providing patient-centered care to culturally diverse patients [76]. Still others notice an evolution in cultural competence, from a multicultural approach that is categorical, subject to stereotypical thinking and one that does not lead to clinical competence, to a cross-cultural approach for which the basis is a patient-centered approach [77]. Our review, providing the patient’s perspective, adds to the theory that patient-centered care is the fundamental basis in providing diversity-sensitive care.

Perceived lack of mutual understanding and negative experiences regarding certain competencies in the context of patient-centered care and diversity-sensitive care in some cases lead to a clear preference for patient and healthcare provider concordance. In literature, a call for concordant care is not universally expressed. For instance, in research on the patient’s perspective of diversity-sensitive care focussing on gender and sexual identity, patients’ reports lack desire for concordance [78]. Some patients believe that the same level of understanding can be attained even without concordance, through principles such as equality and mutual respect [69]. On this basis, it remains unclear if there are discrepancies in preferences for concordant care between social determinants and what the underlying mechanisms for these discrepancies could be. Furthermore, emerging evidence on the effectiveness of concordance is somewhat inconsistent. Some studies show promising results, namely a positive relationship between patient and healthcare provider concordance and reported patient experiences and outcomes [79, 80]. Other studies report positive effects for (only) language concordance [81, 82] and still others show inconclusive results and point out the need for further research [83, 84]. In conclusion, the academic debate on concordant care is still ongoing. Next to this, providing concordant care poses a huge practical challenge. Whatever the outcomes of concordant care, the lack of diversity in healthcare staff should be actively addressed in order to achieve a realistic representation of society in the composition of healthcare providers and students [83].

### Strengths and limitations

This systematic review has limitations. A first limitation is that the research team consists only of white researchers. This is a fundamental challenge in diversity research. It can create bias and a lack of perspective [85]. Second, the visual representation of data and the boundaries to each layer create the illusion of delineation and linear improvement, while the reality could be more diffuse and the placement of competencies into categories is subject to interpretation. However, through extensive deliberation with the research team we aimed to counter these limitations and provide perspective and valid interpretations. Moreover, due to limitations in time and resources, no random sample of test screening for consistency was performed during the screening process. Further, the definition of diversity, vulnerable patient groups and intersectionality made deciding which patient determinants to involve in our search difficult. The lack of clarity of definitions in research on diversity in healthcare impacts results and comparability of different approaches. Also, the geographical and societal context of research influences both determinants and results.

Furthermore, some of the current criticism towards cultural competence pertains to its inability to cover systemic mechanisms and power imbalances that cultural safety / cultural humility does cover [17]. This review was designed to investigate the patient perspective on diversity-sensitive care, primarily focusing on the interpersonal relationship as an initial investigation point. Expanding the scope of this review to encompass systemic mechanisms would have been unfeasible. Therefore, systemic mechanisms were not included in the search syntax. Consistently, throughout the results we noted that patients’ reports in the studies mainly focus on individual competencies. The extent to which systemic mechanisms influence the patient’s perspective of diversity-sensitive care remains unclear and requires further research.

Conversely, the study possesses significant strengths that enhance its validity. Multiple efforts were undertaken to ensure the robustness of this study. A comprehensive and thorough search strategy was employed to identify all relevant articles. The involvement of a methodologist and biomedical information specialist ensured the quality of the search strategy and overall methods. The study adhered closely to the guidelines set forth by Cochrane and PRISMA. Furthermore, a mixed-methods design was utilized, enabling the combination of qualitative and quantitative data. This approach strengthened the study's ability to provide a comprehensive and robust answer to the research questions at hand. By incorporating multiple research methodologies, a more nuanced and comprehensive understanding of the topic was achieved.

### Clinical practice

As patient-centered care is perceived not only as fundamental for diversity-sensitive care, but also as inadequate to this day, healthcare providers must be mindful of the significance of these competencies. In the complexity of diversity-sensitive care, basic medical consultation must not be overlooked. More so, given the experiences, it deserves more attention and training. Reported positive experiences demonstrate the opportunity to grow in these competencies through training and awareness. The ability of training in diversity-sensitive care in improving ethnic minority patient satisfaction has been well documented [86].

### Further research

This systematic review shows many opportunities for further research. First, as this study has focused on healthcare provider’s individual competencies, the patient perspective on diversity-sensitive care at a system level is not sufficiently acknowledged and investigated. The aspect of systemic mechanisms and power imbalances that cultural safety incorporates is scarcely mentioned by patients in our review. This could arguably be due to our focus on individual competencies, and should be further investigated. Second, by pooling patients from very diverse backgrounds, we saw possible differences in experiences and expectations between patient groups. It may be interesting to elaborate on these differences. Lastly, we also pooled all patient – healthcare provider relationships. Further research could investigate whether differences in the patient’s perspective arise between e.g. a medical relationship, a therapeutic relationship or a care relationship.

## Conclusion

The patient perspective on diversity-sensitive care can be summarized in three components: patient-centered care, diversity-sensitive care and patient-provider concordant care. To achieve more diversity-sensitive care, healthcare providers should adopt competencies: (1) attitudes (demonstration of respect, concern, self-awareness, community partnership, allyship) and (2) skills (communication, individualized care, shared decision making, adjustment of care to cultural needs, providing culturally tailored information) and (3) knowledge (cultural, religic and spiritual knowledge). Patients experience inequalities, problems and shortcomings across patient-centered care and diversity-sensitive care competencies, even with the most basic and obvious ones such as respect, concern and attention. These adverse experiences represent a critical motivating factor for patients in expressing a preference for concordant care, specifically with regard to linguistic, ethnic, or gender concordance, in order to experience more diversity-sensitive care.

## Data Availability

No datasets were generated or analysed during the current study.

## Appendix 1

Appendix 1 contains detailed information about the search strategy and the database searching.

**Table:**

Database searched	Platform	Years of coverage	Records	Records after duplicates removed
Medline ALL	Ovid	1946—Present	2016	2006
Embase	Embase.com	1971—Present	2496	796
Web of Science Core Collection^a^	Web of Knowledge	1975—Present	2371	752
Cochrane Central Register of Controlled Trials^b^	Wiley	1992—Present	123	15
CINAHL^c^	EBSCO	1982—Present	1267	248
PsycINFO	Ovid	1806—Present	2114	1377
Additional Search Engines: Google Scholar^d^			200	163
**Total**			**10,587**	**5357**

^a^Science Citation Index Expanded (1975-present); Social Sciences Citation Index (1975-present); Arts & Humanities Citation Index (1975-present); Conference Proceedings Citation Index- Science (1990-present); Conference Proceedings Citation Index- Social Science & Humanities (1990-present); Emerging Sources Citation Index (2005-present)

^b^Manually deleted 62 abstracts from trial registries

^c^Limited to Academic Journals

^d^Google Scholar was searched via "Publish or Perish" to download the results in EndNote

No other database limits were used than those specified in the search strategies

### Medline

(Transcultural Nursing / OR Cultural Competency / OR ((Anthropology, Cultural /) AND (Professional Competence /)) OR (((transcultur* OR trans-cultur* OR crosscultur* OR cross-cultur* OR multicultur* OR multi-cultur*) ADJ3 (care* OR healthcare* OR primary health care OR hospital* OR psychiatr* OR nurs* OR service* OR training* OR orient* OR expert* OR skill* OR behav* OR acceptabilit* OR humilit* OR service* OR communicat* OR barrier* OR background* OR belief* OR value*)) OR ((cultur* OR intercultur* OR diversit*) ADJ6 (appropriat* OR sensitiv* OR congruent* OR competen* OR tailor*))).ab,ti,kw.) AND (Patient Satisfaction / OR (Personal Satisfaction / AND patient/) OR ((patient* OR client* OR indigenous* OR aboriginal* OR immigrant* OR migrant* OR refugee* OR minorit* OR ethnic*) ADJ3 (perspective* OR expectation* OR experience* OR satisf* OR perception* OR perceiv*)).ab,ti,kw.) NOT (exp * Education/ OR (education* OR training*).ti.)

### embase.com

('transcultural care'/exp OR 'cultural competence'/de OR 'cultural sensitivity'/de OR (('cultural factor'/de OR 'cultural anthropology'/de) AND ('professional competence'/de)) OR (((transcultur* OR trans-cultur* OR crosscultur* OR cross-cultur* OR multicultur* OR multi-cultur*) NEAR/3 (care* OR healthcare* OR 'primary health care' OR hospital* OR psychiatr* OR nurs* OR service* OR training* OR orient* OR expert* OR skill* OR behav* OR acceptabilit* OR humilit* OR service* OR communicat* OR barrier* OR background* OR belief* OR value*)) OR ((cultur* OR intercultur* OR diversit*) NEAR/6 (appropriat* OR sensitiv* OR congruent* OR competen* OR tailor*))):ab,ti,kw) AND ('patient perspective'/de OR 'patient experience'/de OR 'patient expectation'/de OR 'patient satisfaction'/de OR ('personal experience'/de AND patient/exp) OR ((patient* OR client* OR indigenous* OR aboriginal* OR immigrant* OR migrant* OR refugee* OR minorit* OR ethnic*) NEAR/3 (perspective* OR expectation* OR experience* OR satisf* OR perception* OR perceiv*)):ab,ti,kw) NOT [conference abstract]/lim NOT (education/mj OR (education* OR training*):ti).

### Web of science

TS = (((((transcultur* OR trans-cultur* OR crosscultur* OR cross-cultur* OR multicultur* OR multi-cultur*) NEAR/2 (care* OR healthcare* OR "primary health care" OR hospital* OR psychiatr* OR nurs* OR service* OR training* OR orient* OR expert* OR skill* OR behav* OR acceptabilit* OR humilit* OR service* OR communicat* OR barrier* OR background* OR belief* OR value*)) OR ((cultur* OR intercultur* OR diversit*) NEAR/5 (appropriat* OR sensitiv* OR congruent* OR competen* OR tailor*)))) AND (((patient* OR client* OR indigenous* OR aboriginal* OR immigrant* OR migrant* OR refugee* OR minorit* OR ethnic*) NEAR/2 (perspective* OR expectation* OR experience* OR satisf* OR perception* OR perceiv*)))) NOT TI = ((education* OR training*)) NOT DT = (Meeting Abstract OR Meeting Summary) AND LA = (english).

### Cochrane

((((transcultur* OR trans NEXT cultur* OR crosscultur* OR cross NEXT cultur* OR multicultur* OR multi NEXT cultur*) NEAR/3 (care* OR healthcare* OR 'primary health care' OR hospital* OR psychiatr* OR nurs* OR service* OR training* OR orient* OR expert* OR skill* OR behav* OR acceptabilit* OR humilit* OR service* OR communicat* OR barrier* OR background* OR belief* OR value*)) OR ((cultur* OR intercultur* OR diversit*) NEAR/6 (appropriat* OR sensitiv* OR congruent* OR competen* OR tailor*))):ab,ti) AND (((patient* OR client* OR indigenous* OR aboriginal* OR immigrant* OR migrant* OR refugee* OR minorit* OR ethnic*) NEAR/3 (perspective* OR expectation* OR experience* OR satisf* OR perception* OR perceiv*)):ab,ti) NOT ((education* OR training*):ti).

### CINAHL

(MH Transcultural Nursing OR MH Cultural Competence OR ((MH Anthropology, Cultural) AND (MH Professional Competence)) OR TI(((transcultur* OR trans-cultur* OR crosscultur* OR cross-cultur* OR multicultur* OR multi-cultur*) N2 (care* OR healthcare* OR primary health care OR hospital* OR psychiatr* OR nurs* OR service* OR training* OR orient* OR expert* OR skill* OR behav* OR acceptabilit* OR humilit* OR service* OR communicat* OR barrier* OR background* OR belief* OR value*)) OR ((cultur* OR intercultur* OR diversit*) N5 (appropriat* OR sensitiv* OR congruent* OR competen* OR tailor*))) OR AB(((transcultur* OR trans-cultur* OR crosscultur* OR cross-cultur* OR multicultur* OR multi-cultur*) N2 (care* OR healthcare* OR primary health care OR hospital* OR psychiatr* OR nurs* OR service* OR training* OR orient* OR expert* OR skill* OR behav* OR acceptabilit* OR humilit* OR service* OR communicat* OR barrier* OR background* OR belief* OR value*)) OR ((cultur* OR intercultur* OR diversit*) N5 (appropriat* OR sensitiv* OR congruent* OR competen* OR tailor*)))) AND (MH Patient Satisfaction OR (MH Personal Satisfaction AND MH patient) OR TI((patient* OR client* OR indigenous* OR aboriginal* OR immigrant* OR migrant* OR refugee* OR minorit* OR ethnic*) N2 (perspective* OR expectation* OR experience* OR satisf* OR perception* OR perceiv*)) OR AB((patient* OR client* OR indigenous* OR aboriginal* OR immigrant* OR migrant* OR refugee* OR minorit* OR ethnic*) N2 (perspective* OR expectation* OR experience* OR satisf* OR perception* OR perceiv*))) NOT (MM Education + OR TI(education* OR training*)).

### PsycINFO

(Transcultural Psychiatry / OR Cross Cultural Treatment / OR Cultural Sensitivity / OR (("Culture (Anthropological)" / OR Cross Cultural Differences / OR Cross Cultural Psychology /) AND (Professional Competence /)) OR (((transcultur* OR trans-cultur* OR crosscultur* OR cross-cultur* OR multicultur* OR multi-cultur*) ADJ3 (care* OR healthcare* OR psychiatr* OR nurs* OR service* OR training* OR orientat*)) OR ((cultur* OR intercultur* OR diversit*) ADJ6 (appropriat* OR sensitiv* OR congruent* OR competen* OR tailor*))).ab,ti.) AND (Client Satisfaction / OR (Satisfaction / OR exp Patients/) OR ((patient* OR client* OR indigenous* OR aboriginal* OR immigrant* OR minorit* OR ethnic* OR resident*) ADJ3 (perspective* OR expectation* OR experience* OR satisf* OR perception* OR perciev*)).ab,ti.) AND english.la.

### Google scholar

"transcultural|crosscultural|multicultural care|healthcare|health"|"trans|cross|multi cultural care|healthcare|health" "patient|client|resident perspective|expectations|experiences|satisfaction|perceptions".

## Appendix 2

Appendix 2 contains detailed information about the quality assessment.
